# Awareness of performance on outcomes after total hip and knee arthroplasty among Dutch orthopedic surgeons: how to improve feedback from arthroplasty registries

**DOI:** 10.1080/17453674.2020.1827523

**Published:** 2020-10-06

**Authors:** Peter Van Schie, Leti Van Bodegom-Vos, Tristan M Zijdeman, Rob G H H Nelissen, Perla J Marang-Van De Mheen

**Affiliations:** a Department of Orthopedics, Leiden University Medical Centre, Leiden;; b Department of Biomedical Data Sciences, Medical Decision Making, Leiden University Medical Centre, Leiden, the Netherlands

## Abstract

Background and purpose — The Netherlands Registry of Orthopedic Implants (LROI) uses audit and feedback (A&F) as the strategy to improve performance outcomes after total hip and knee arthroplasty (THA/TKA). Effectiveness of A&F depends on awareness of below-average performance to initiate improvement activities. We explored the awareness of Dutch orthopedic surgeons regarding their performance on outcomes after THA/TKA and factors associated with this awareness.

Methods — An anonymous questionnaire was sent to all 445 eligible Dutch orthopedic surgeons performing THA/TKA. To assess awareness on own surgeon-group performance, they were asked whether their 1-year THA/TKA revision rates over the past 2 years were below average (negative outlier), average (non-outlier), above average (positive outlier) in the funnel plot on the LROI dashboard, or did not know. Associations were determined with (1) dashboard login at least once a year (yes/no); (2) correct funnel-plot interpretation (yes/no) and; (3) recall of their 1-year THA/TKA revision rate (yes/no).

Results — 44% of respondents started the questionnaire, 158 THA and 156 TKA surgeons. 55% of THA surgeons and 55% of TKA surgeons were aware of their performance. Surgeons aware of their performance more often logged in on the LROI dashboard, more often interpreted funnel plots correctly, and more often recalled their revision rate. 38% of THA and 26% of TKA surgeons scored “good” on all 3 outcomes.

Interpretation — Only half of the orthopedic surgeons were aware of their performance status regarding outcomes after THA/TKA. This suggests that to increase awareness, orthopedic surgeons need to be actively motivated to look at the dashboard more frequently and educated on interpretation of funnel plots for audit and feedback to be effective.

Several studies have shown large between-hospital variation in performance outcomes after total hip and knee arthroplasty (THA/TKA) including revision rates, suggesting opportunities to improve care (Siciliani et al. 2013, Bozic et al. 2014, Menendez et al. 2016, Weeks et al. 2016, Fry et al. 2017, van Schie et al. 2020). Audit and feedback (A&F) is a frequently used approach to reduce between-hospital variation, and defined as provision of clinical performance summaries to healthcare providers or organizations intended to initiate activities to improve performance (Brehaut and Eva 2012, Ivers et al. 2014). Worldwide, A&F from arthroplasty registries is provided in different ways. In the Netherlands, performance indicators such as revision rates, patient-reported outcome measures (PROMs), and patient characteristics are shown on surgeon-group level in a real-time password-protected web-based dashboard and the extent of variation is shown in an anonymized version in annual reports.

Following a Cochrane review of 140 studies from multiple fields, A&F is effective with a median absolute improvement of 4% of the desired outcome, but with the effect size varying from a 9% decrease to a 70% increase (Ivers et al. 2012). Part of the reason for this large variation in effectiveness may be the varying degree to which A&F leads to an increased awareness of own performance. For example, A&F is not received, information including graphs (e.g., funnel plots) and/or tables is not interpreted correctly, or the reported performance outcomes are not considered interesting (Gude et al. 2018). Sufficient awareness of own performance relative to others in combination with motivation to improve is more likely to result in targeted quality improvement initiatives (Davis et al. 2006, van der Veer et al. 2010, de Vos Maartje et al. 2013).

Due to a lack of awareness of own performance, it is often overestimated (Gude et al. 2018). This can limit quality improvement initiatives, because it is assumed that performance is good even though there may be room for improvement. Furthermore, it is important that performance indicators give sufficient direction on where to improve care, so that professionals are able to select focused interventions to improve care. A recent study showed that for most surgeon groups with significantly higher revision rates, the direction of improvement could be pointed out by looking at the reason for revision (e.g., infection, prosthesis loosening, dislocation etc.) (van Schie et al. 2020). By looking at a more specific outcome, professionals can figure out in which part of the care process improvements are possible, e.g., timing of antibiotic prophylaxis (infection), cementation techniques (prothesis loosening), or femoral head size (dislocation).

We explored the awareness of orthopedic surgeons regarding their performance on outcomes after THA/TKA and factors associated with this awareness, to gain insight into the ways to increase the effectiveness of A&F provided by the LROI.

## Methods

An anonymous internet-based questionnaire study was performed in December 2018 to explore the awareness of orthopedic surgeons on outcomes after THA/TKA provided by the LROI and associated factors.

### Netherlands Registry of Orthopedic Implants (LROI)

The LROI was established in 2007 and in 2012 all Dutch surgeon groups participated. In 2015, the LROI dashboard was developed to allow surgeons to better monitor their performance showing information on the number of procedures performed, revision rates, PROMs and patient characteristics on surgeon-group level compared with other surgeon groups, which can be viewed at any time. The completeness for primary THA and TKA procedures is checked against Electronic Health Records and is currently above 98% for primary procedures and 96% for revisions (van Steenbergen et al. 2015). 97 surgeon groups performed THA and 98 performed TKA in the study period.

### Study population

The questionnaire was sent to all 445 Dutch orthopedic surgeons performing primary THA/TKA who were members of the hip and knee working groups from the Dutch Orthopedic Association. Reminders were sent by email 4 and 8 weeks after the first invitation. The survey was compiled using NetQ software (version 2014.Q3; https://cumulusnetworks.com/products/netq/).

### Survey

The information collected with the survey regarding the feedback provided on the LROI dashboard is divided into 4 parts (Appendix, see Supplementary data).

In the first part, awareness regarding possible deviating performance (outlier status) of their own surgeon group over the last 2 years was assessed by asking whether their 1-year revision rate was below average (negative outlier), average (non-outlier), above average (positive outlier), in the funnel plot on the LROI dashboard, or they did not know. Second, we searched for 3 potential underlying factors that might be related to the level of awareness. It was assessed whether respondents (1) logged in at least once a year on their LROI dashboard; (2) were able to interpret funnel plots correctly; (3) could recall the 1-year revision rate of their surgeon group. Respondents answering that they did not know were counted as giving a non-positive answer. By combining these 3 questions, a composite outcome was created. A respondent only scored “good” when all 3 individual measures were positive, i.e., he/she logged in at least once a year, correctly interpreted the funnel plots, and could recall his/her 1-year revision rate. We also asked about hospital work setting (university/teaching/general hospital or private clinic) and number of arthroplasties performed annually (< 50, 50–100, > 100). Third, respondents were asked about quality improvement initiatives following possible below-average performance (negative outlier) in the past 2 years, and whether the effects of these initiatives were checked using the available feedback information on the LROI dashboard. Finally, there were questions about perceived need for changes in the current feedback, which current performance indicators were considered important, which indicators should be added to improve healthcare and the preferred frequency (every 1, 3, 6, or 12 months) and way of receiving feedback (tailored for their surgeon group or ability to make selections and explore the data oneself).

### Statistics

Analyses were performed separately for THA and TKA surgeons. First, the proportion of respondents who were aware of deviating performance for their own surgeon group in the past 2 years was assessed. To examine the associations between awareness of deviating performance and the predefined potentially underlying factors (login to the dashboard, correct interpretation of funnel plots, recall of their own revision rate), univariate logistic regression analyses were performed. All questions answered by respondents regardless of whether they completed the full survey were included in the analyses. If surgeons stopped the survey but answered the previous question, we assumed there was a reason for stopping at that specific question (e.g., because it would be not acceptable to say not logging in) and coded this question as “don’t know,” meaning these were included as non-positive answers. In addition, we examined whether the composite outcome differed across hospital settings and number of THAs/TKAs performed annually.

Data were analyzed with the statistical software SPSS version 25 (IBM Corp, Armonk, NY, USA). P-values < 0.05 were considered statistically significant in all analyses.

### Ethics, funding, and potential conflicts of interest

The LUMC Medical Ethical Committee waived the need for ethical approval under Dutch law (CME, G18.140). Author PvS received a grant from the Van Rens Foundation (VRF2018-001) to perform this study. The authors declare that there are no conflicts of interest.

## Results

**Figure 1.**
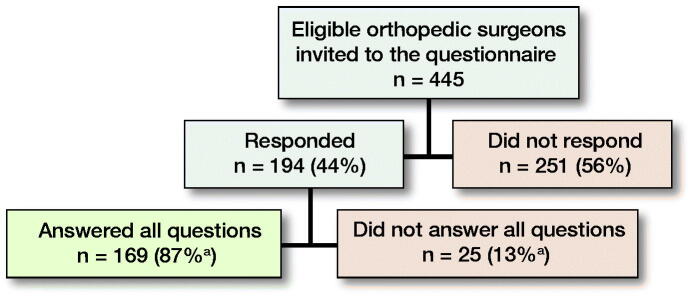
Respondent flowchart. **^a^** Percentage of total number of respondents.

From 445 invited orthopedic surgeons, 194 (44%) started the survey; 158 surgeons performed THA and 156 TKA. 78 answered the questions within 4 weeks, 56 after the 1st, and 60 after the 2nd reminder. 169 (87%) respondents completed the survey (Figure 1). Median time to complete the survey was 6.4 minutes (interquartile range 5.3–8.5).

91% of respondents were male and 52% were between 40 and 50 years old. Most respondents (40%) were employed in a general hospital and evenly distributed across volume groups for THA and TKA (Table 1).

**Table 1.** Characteristics of the respondents (n = 194). Values are frequency (%)

Factor	n (%)
Male sexle	177 (91)
Age	
	32 (16)
40–50	101 (52)
51–60	42 (22)
	19 (10)
Hospital setting	
University medical center	20 (10)
Teaching hospital	72 (37)
General hospital	78 (40)
Private clinic	24 (13)
Type of surgeon **^a^**	
Performing THA	158 (81)
Performing TKA	156 (80)
Performing THA and TKA	120 (62)
No. of THAs/surgeon/year **^b^**	
	34 (21)
50–100	75 (48)
	46 (29)
No response	3 (2)
No. of TKAs/surgeon/year **^c^**	
	37 (24)
50–100	78 (50)
	32 (20)
No response	9 (6)

**^a^** Does the respondent perform only THA,

only TKA, or both THA and TKA?

**^b^** There were 158 THA surgeons.

**^c^** There were 156 TKA surgeons.

**Table 2.** Associations between awareness of surgeon-group performance and logging in to dashboard, correct funnel-plot interpretation, and knowledge of 1-year revision rate

All respondents performing	Logging on to LROI dashboard ^a^		Correct funnel-plot interpretation ^b^		Knowledge of 1-year revision rate ^c^	
	Yes, n (%)	OR (CI) ^d^	Yes, n (%)	OR (CI) ^d^	Yes, n (%)	OR (CI) ^d^
THA (n = 158)	105 (67)		96 (61)		105 (67)	
Aware of surgeon-group performance						
Yes (n = 77)	69 (90)	7.6 (3.2–18)	59 (77)	2.4 (1.2–4.9)	66 (86)	4.4 (2.0–9.8)
No (n = 64)	34 (53)	Reference	37 (58)	Reference	37 (58)	Reference
TKA (n = 156)	103 (66)		95 (61)		103 (66)	
Aware of surgeon-group performance						
Yes (n = 78)	65 (83)	4.1 (1.9–9.0)	56 (72)	1.6 (0.8–3.3)	66 (85)	4.9 (2.2–11)
No (n = 64)	35 (55)	Reference	39 (61)	Reference	34 (53)	Reference

**^a^** Logging in at least once every year.

**^b^** Correctly interpreted both funnel plots.

**^c^** Know the 1-year revision rate of their healthcare center of the past 2 years.

**^d^** OR (CI) = odds ratio (95% confidence interval).

**Table 3.** Composite outcome, stratified by hospital setting and number of arthroplasties performed annually

	Composite outcome good, n	Odds ratio
THA surgeons		
All THA-performing respondents (n = 158)	60	
Aware of surgeon-group performance		
Yes (n = 77)	46	5.3
No (n = 64)	14	Ref.
Hospital setting (n = 158)		
University medical center	4	0.4
Teaching hospital	19	0.6
General hospital	31	Ref.
Private clinic	6	0.7
No. of THAs performed per year (n = 155) ^a^		
	7	0.4
50–100	35	1.4
	18	Ref.
TKA surgeons		
All TKA-performing respondents (n = 156)	41	
Aware of surgeon-group performance:		
Yes (n = 78)	31	3.6
No (n = 64)	10	Ref.
Hospital setting (n = 156)		
University medical center	2	0.3
Teaching hospital	15	0.6
General hospital	23	Ref.
Private clinic	1	0.1
No. of TKAs performed per year (n = 147) ^a^		
	7	2.4
50–100	28	1.0
	6	Ref.

**^a^** Number of arthroplasties performed per year by the respondent.

### Awareness of performance and underlying factors (Tables 2 and 3)

158 THA surgeons answered the questions on logging in, funnel-plot interpretation, and recalling their revision rate. Only 141 THA surgeons answered the questions on awareness of their surgeon-group performance, with 77 (55%) THA surgeons indicating awareness of any deviating performance in their surgeon group over the past 2 years. Among the 158 THA surgeons, 105 (67%) logged in on the LROI dashboard at least once a year, 96 (61%) interpreted the funnel plot correctly, and 105 (67%) recalled their 1-year revision rate. THA surgeons who were aware of any deviating performance were 8 times more likely to log in, twice as likely to correctly interpret the funnel plot, and 4 times more likely to recall their 1-year revision rate. Overall, 66 (38%) respondents scored “good” on all these individual items and thus on the composite outcome. THA surgeons who are aware of deviating performance were 5 times more likely to score “good” on the composite outcome.

156 TKA surgeons answered the questions on logging in, funnel-plot interpretation, and recalling their revision rate. Only 142 TKA surgeons answered the questions on awareness of own surgeon-group performance, with 78 (55%) TKA surgeons indicating awareness of any deviating performance in their surgeon group over the past 2 years. Among the 156 TKA surgeons, 103 (66%) logged in to the LROI dashboard at least once a year, 95 (61%) interpreted the funnel plot correctly, and 103 (66%) recalled their 1-year revision rate. TKA surgeons who were aware of any deviating performance were 4 times more likely to log in, twice as likely to correctly interpret the funnel plot, and 5 times more likely to recall their 1-year revision rate. Overall, 41 (26%) respondents scored “good” on the composite outcome and TKA surgeons who are aware of deviating performance were 4 times more likely to score “good” on the composite outcome.

The proportion of surgeons who met the criteria of the composite outcome did not differ in the number of arthroplasties performed annually or across hospital settings, except for a lower proportion for TKA surgeons in private clinics.

### Quality improvement initiatives

20 respondents indicated that they were employed in a healthcare center that had a significantly higher 1-year revision rate (negative outlier) in the past 2 years. 9 of them did not see this deviating performance coming, because they had never checked the LROI dashboard for performance indicators. 17 indicated that quality improvement initiatives had been introduced and all of them used performance indicators from the LROI dashboard to monitor the effect. A positive effect of these initiatives on the revision rate was reported by 9 respondents and a negative effect by 3 respondents when checking progress in the LROI dashboard. 5 respondents were currently following the effect.

### Future feedback

**Figure 2.**
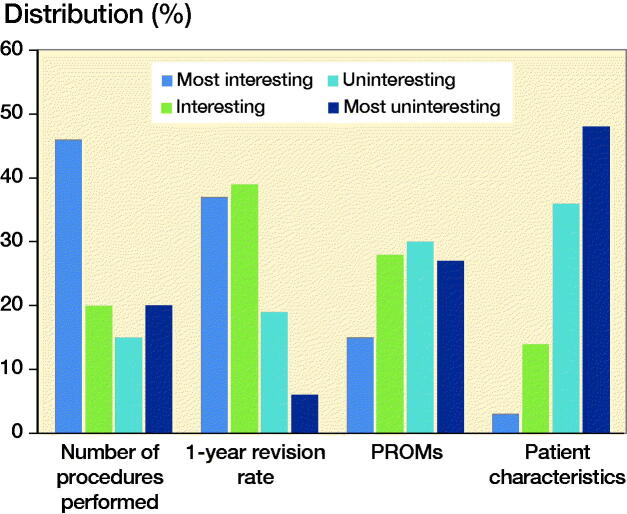
Currently available performance indicators on the secure LROI dashboard ranked from most to least interesting by respondents. LROI = Netherlands Registry of Orthopedic Implants; PROMs = patient-reported outcome measures.

From the current available performance indicators, the number of procedures performed was mostly considered as the most interesting information on the LROI dashboard, followed by 1-year revision rates, PROMs, and patient characteristics respectively (Figure 2).

**Figure 3.**
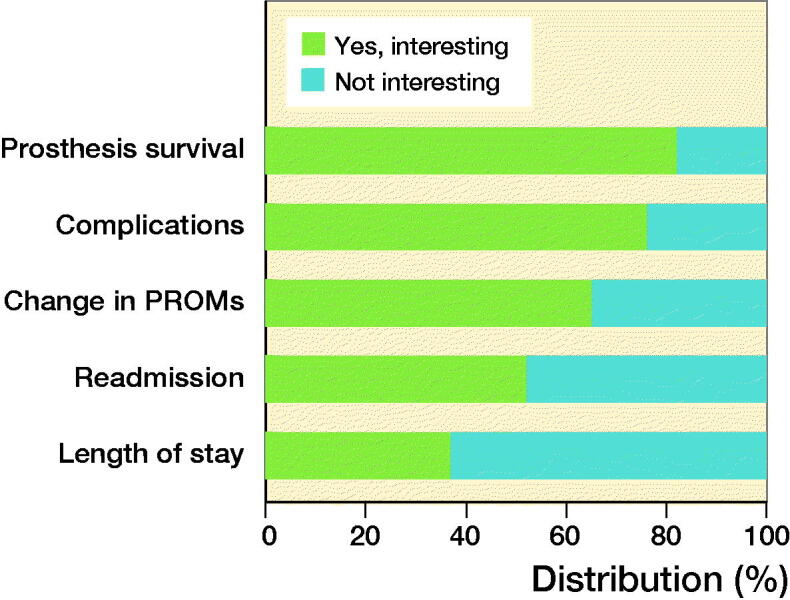
Percentage of orthopedic surgeons interested in additional performance indicators. Change = difference between pre- and postoperative PROMs. For abbreviations, see Figure 2

Prosthesis survival and complications are currently not available on the LROI dashboard, but 138 (82%) THA surgeons and 129 (76%) TKA surgeons indicated this information to constitute relevant indicators (Figure 3). 106 (62%) respondents would prefer to receive feedback every 6 months, and a minority every month (n = 6, 4%), or every quarter (n = 40, 23%), with some respondents having no preference (n = 18, 11%). 139 (82%) respondents prefer feedback that is tailored to their surgeon group without making any selections and 30 respondents (18%) indicated preferring to make their own selections of LROI indicators.

## Discussion

Although Dutch orthopedic surgeons performing THA/TKA can view their surgeon-group performance on a web-based A&F dashboard, only half of them are actually aware of their performance over the past 2 years. This lack of awareness of own performance and the associations found in our study suggests that orthopedic surgeons need to be actively motivated to log in more often, need to be educated on how to interpret funnel plots correctly, and must be able to reproduce their revision rate for the A&F to be effective in improving care. To act on the feedback information all underlying factors must be met, but this was the case in only one-third of THA surgeons and one-fourth of TKA surgeons and was fairly similar across different types of hospitals and annual volume. Yet, it seems important to increase the effectiveness of feedback, given that 9 out of 20 respondents in the negative outlier surgeon groups indicated that they did not see their worsening performance coming. Without effective feedback, surgeon groups would continue to provide care without modification, while 17 of these 20 respondents indicated that they conducted quality improvement initiatives once identified as showing poor performance.

### Differences and similarities between national arthroplasty registries in providing A&F

The way in which A&F is offered varies, from publicly available annual reports including only nationwide averages with sometimes additional surgeon-group-specific performance, whereas others publish their indicators on surgeon-group level and surgeon level only in password-protected online dashboards (Li et al. 1999, Itonaga et al. 2000, Tabak et al. 2002, Bonutti et al. 2017, Kurcz et al. 2018, Talmo et al. 2018, Assi et al. 2019, Pelt et al. 2019, Porter et al. 2019, Yoon et al. 2019). The LROI, National Joint Registry in the United Kingdom (NJR), and Swedish Hip Arthroplasty Registries (SHAR) use a web-based password-protected A&F dashboard to provide surgeons with peer-comparison indicators in visual graphs on surgeon-group level and in the United Kingdom also on surgeon level (Toomey et al. 2001, Tabak et al. 2002, Assi et al. 2019, Porter et al. 2019, Yoon et al. 2019). In contrast, the Swedish Knee Arthroplasty Registries (SKAR) and the Danish Hip Arthroplasty Registries (DHAR) make no use of online dashboards, where the SKAR publishes only some indicators (e.g., patient demographics and PROMs) on its publicly accessible website once a year. Some arthroplasty registers may inform participating hospitals once a year about their performance, e.g., by emailing performance indicators without this being listed on their website. The feedback generated by the NJR is updated every 6 months, which was also indicated as the preferred frequency to receive feedback by two-thirds of respondents in our study (Porter et al. 2019). The Finnish Arthroplasty Register (FAR) even uses a daily updated publicly accessible website, which includes patient demographics and revision rates at surgeon-group level (Tabak et al. 2002). What all these different methods of feedback have in common is that it is passive education not requiring any action, which may be one of the explanations for orthopedic surgeons being unaware of their performance. Public availability of performance indicators may increase the likelihood of action being taken, given that both patients and other stakeholders such as insurance companies can review the data and may use them in their decision-making.

### Comparison with literature

Besides the Cochrane Review, there are more studies that found wide variation in the effect of A&F (Ivers et al. 2012). A review evaluating interactive computer feedback found a highly variable effect of improvement in quality of care in 3 out of 7 studies (Tuti et al. 2017). Another more recent study found a significant improvement for 4 out of 6 performance indicators, 2.5 years after implementation of online A&F interventions in maternal-newborn hospitals (Weiss et al. 2018). Given the varying effect of A&F, the results of our study can make a relevant contribution to further improve current feedback as provided by arthroplasty registries. We have gained insight into whether A&F reached the target group (i.e., how often surgeons log in), the ability to interpret the funnel plot, and recall of revision rates. In addition, we investigated which performance indicators currently provided by the LROI are considered important by the target group and which indicators should be added. Furthermore, it would be useful to provide feedback on the reasons for revisions, given that this has been shown able to direct quality improvement initiatives although we did not specifically ask whether orthopedic surgeons would be interested in this information (van Schie et al. 2020). 2 meta-analyses have shown that a single A&F strategy is one of the less effective interventions showing little to no improvement when examined (Shojania et al. 2006, Tricco et al. 2012). On the other hand, it seems obvious that accessible A&F that is interpreted correctly will ultimately improve the quality of care, as 17 out of 20 orthopedic surgeons indicated that they would conduct quality improvement initiatives as soon as they become aware of poorer performance. It seems likely that more active elements need to be added both to motivate orthopedic surgeons to log in and to ensure correct interpretation of the funnel plot, which is needed to be aware of outlier status regarding their performance.

Trust in A&F data quality is often identified as a barrier to change clinical behavior. This is unlikely to play a major role in the current LROI feedback given the 98% completeness for primary procedures and 96% for revisions, which is similar for the data in the above-mentioned arthroplasty registries (van der Veer et al. 2010, de Vos Maartje et al. 2013, van Steenbergen et al. 2015, Catelas et al. 2018, Gude et al. 2018). Another barrier may be that physicians do not consider some indicators as an essential part of quality or deem benchmarks unrealistic (van der Veer et al. 2011, Eva et al. 2012, Gude et al. 2016, Gude et al. 2017b, Gude et al. 2018). In this study, for instance, it was found that one-third of both THA and TKA surgeons do not know their 1-year revision rate, which may suggest that some surgeons do not recognize the importance of this outcome. This is striking because this outcome is already widely used by arthroplasty registries and considered an indicator to reflect the quality of care (Li et al. 1999, Itonaga et al. 2000, Tabak et al. 2002, Bonutti et al. 2017, Talmo et al. 2018). Moreover, A&F does not use absolute benchmarks, but performance indicators are compared with national surgeon-group averages, thereby making it likely that other similar surgeon groups are able to achieve that level of performance.

### Strengths and limitations

A possible limitation of this study is response bias if awareness of performance differs between responders and non-responders and the association with underlying factors were to be different. Given that survey responses were collected anonymously, we were unable to compare whether the characteristics of the non-respondents differed from the respondents to assess whether bias may have occurred. However, considering the overall response rate of 44%, and the fact that non-respondents in general are not as involved as respondents and are thus more likely not to be aware of their performance, the associations are likely underestimated. A second limitation is that some self-reported outcomes (e.g., frequency of logging in or recall of revision rate) were analyzed. It is therefore possible that there were socially desirable answers to certain questions, e.g., knowledge about certain indicators. If this affected the results, even fewer orthopedic surgeons may be aware of their performance. However, because this was an anonymous survey, it seems more likely that respondents are surgeons dedicated to good performance and making feedback information more useful rather than giving socially desirable answers, so that reported rates are likely to reflect actual practice. An exception on the self-reported outcomes was the funnel-plot interpretation, where answers given by respondents were compared with the correct answer so that social desirability was not an issue. A third limitation may be the generalization of our results to other countries. Increasingly, information becomes publicly available on differences between hospitals in patient outcomes, as we have previously shown for revision rates in the Netherlands and Bozic et al. (2014) have shown for complication rates after total hip and knee arthroplasty in the United States (Bozic et al. 2014, van Schie et al. 2020). The magnitude of the between-hospital variation in risk-adjusted rates in these studies is surprisingly similar, with both studies showing about 3–4-fold differences between hospitals. Furthermore, although not looking at awareness in performance specifically, a previous international survey study showed only minor differences between orthopedic surgeons operating on different continents, taking into account their demographics (e.g., sex, age), surgical experience (e.g., number of years in practice, number of arthroplasties performed per year), use of additional diagnostics (e.g., plain radiographs, CT, MRI), and final treatment chosen (e.g., surgical versus non-surgical) (Li et al. 2014). So there is no evidence to suggest that there would be smaller differences between surgeons regarding their performance in other countries, and a difference in awareness has to our knowledge not been described before. Yet, such difference in awareness may be crucial in explaining why hospital differences in performance continue to exist, rather than that public reporting of hospital differences will by itself result in improvement.

### Implementation and further research

As alluded to earlier, more active elements need to be added to improve A&F design to make it more attractive to log in, resulting in more awareness of one’s own performance. This could be encouraged by emphasizing the importance of already available indicators (e.g., revision rates) and adding new indicators to the A&F dashboard that are considered relevant and of interest as reported in this study (prosthesis survival, complications, readmissions, and length of hospital stay). As a result, more surgeons may be actually reached by the feedback, because the number of orthopedic surgeons who log in as well as the frequency of logging in will then increase. In addition, teaching material must be available on how to interpret funnel plots and be actively promoted by the orthopedic association during meetings, which will also increase awareness and possibly increase the reach of feedback, when more surgeons can interpret the performance indicators. Ultimately, an increased awareness of one’s own performance will likely lead to more quality improvement initiatives.

The question arises as to whether voluntary quality control by providing only passive A&F on performance is sufficient in a modern orthopedic society. A&F could be more effective when offered in a more active and multifaceted way instead of as a single element (which in this study was only the LROI dashboard) (Ivers et al. 2012, Soong and Shojania 2020). A possible addition to the feedback would be that indicators are also verbally explained by an independent person, with clear targets discussed and action plans created, for instance based on a toolbox (Bradley et al. 2004, 2006, de Vos et al. 2009, van der Veer et al. 2010, Ivers et al. 2012, 2014, Brehaut et al. 2016, Gude et al. 2017a, Brown et al. 2019, Roos-Blom et al. 2019). In addition, setting up committees that will actively approach poorly performing hospitals to create action plans to improve quality of care may increase interest in one’s own performance as orthopedic surgeons want to avoid being under supervision. The Dutch Orthopedic Association initiated a quality committee in 2017 with the aim to detect negative outlier hospitals using LROI data and discuss activities to improve care (Commision Quality). This new procedure may stimulate logging in to check on performance and in this way increase awareness of own performance in the coming years. After all, orthopedic surgeons have no valid reason not to be interested in their own performance, given that they want the best care for their patients and continuously improving the quality of care is thus inherently linked to this.

This survey is part of the “Improving Quality based on the Joint registries project” (IQ Joint study). Within this study, what will be tested includes whether more active intervention including monthly feedback on THA/TKA performance indicators, active education on how to use indicators for quality improvement, asking for improvement activities, and linking hospitals with better performing hospitals to exchange information and find areas for improvement will result in better outcomes, fewer complications, and more quality improvement initiatives compared with the LROI dashboard alone. During this randomized trial, A&F on surgeon-group level will be provided according to the preferences of the orthopedic surgeons as has been evaluated in this study.

### Conclusion

Orthopedic surgeons performing THA/TKA have limited awareness of the performance of their surgeon group. Awareness could be increased by encouraging them to log in more often on their A&F dashboard, teaching them how to interpret funnel plots, and emphasizing the importance of performance indicators. Improvement of the effectiveness of feedback is important, because the majority of orthopedic surgeons indicated that quality improvement initiatives were introduced once they learned that their performance was worsening. To provide orthopedic surgeons with better feedback in the future, the feedback information should be extended with the indicators prosthesis survival and complications compared with peers at a national level, tailored to their specific surgeon group rather than making any selections themselves, with 6-month frequency.

## Supplementary Material

Supplemental Material

## Supplementary data

The Appendix is available as supplementary data in the online version of this article, http://dx.doi.org/10.1080/17453674.2020.1827523
