# Supplementary material for: Awareness of performance on outcomes after total hip and knee arthroplasty among Dutch orthopedic surgeons: how to improve feedback from arthroplasty registries
Source: Acta Orthop. 2020 Oct 6;92(1):54–61. doi: 10.1080/17453674.2020.1827523 (PMC7919881; doi:10.1080/17453674.2020.1827523)
Supplement: Supplemental Material [file IORT_A_1827523_SM6886.pdf]

Supplementary data

Appendix: Survey

In order to make the questionnaire run as efficiently as possible, a number of "loops" have been incorporated into the questionnaire, so that orthopedic surgeons do not have to answer questions that do not apply to them. These loops are indicated in the questionnaire.

Questions for all respondents: Surgeon-specific questions

1. What is your age in years?
    a. Under 40
    b. 40 to 50
    c. 51 to 60
    d. Above 60
2. What is your gender?
    a. Male
    b. Female
3. In what type of healthcare center do you work for the majority of your time? (*This question involves part 2, see methods section survey in article*)
    a. University hospital
    b. Teaching hospital
    c. General hospital
    d. Private clinic
4. Do you perform primary total hip arthroplasties and/or primary total knee arthroplasties?
    a. Yes, only hip arthroplasties ➞ Loop I (questions 5, 6, (skip questions 7, 8))
    b. Yes, only knee arthroplasties ➞ Loop II (questions 7, 8 (skip questions 5, 6))
    c. Yes, both hip and knee arthroplasties ➞ Loop I &II (questions 5, 6, 7, 8)
    d. No ➞ End of questionnaire

Loop I: Orthopedic surgeons performing total hip arthroplasties

5. How many primary total hip arthroplasties do you perform annually? (*This question involves part 2, see methods section survey in article*)
    a. Less than 50
    b. 50 to 100
    c. More than 100

Questions regarding the online LROI dashboard for total hip arthroplasty

6. What was the overall 1-year revision rate of your department for total hip arthroplasties over the last 2 years? (*This question involves part 2, see methods section survey in article*)
    a. Worse than average
    b. Average
    c. Better than average
    d. I do not know

Loop II: Orthopedic surgeons performing total knee arthroplasties

7. How many primary total knee arthroplasties do you perform annually? (*This question involves part 2, see methods section survey in article*)
    a. Less than 50
    b. 50 to 100
    c. More than 100

Questions regarding the online LROI dashboard for total knee arthroplasty

8. What was the overall 1-year revision rate of your department for total knee arthroplasties over the last 2 years? (*This question involves part 2, see methods section survey in article)*
    a. Worse than average
    b. Average
    c. Better than average
    d. I do not know

Questions for all respondents: Frequency of logging on to LROI dashboard

9. How often do you log on to the LROI dashboard? (*This question involves part 2, see methods section survey in article*)
    a. Never
    b. Once a week
    c. Once a month
    d. Once per 6 months
    e. Once a year

Questions for all respondents: Funnel-plot interpretation

10. How does the department encircled in red perform? (*This question involves part 2, see methods section survey in article)*

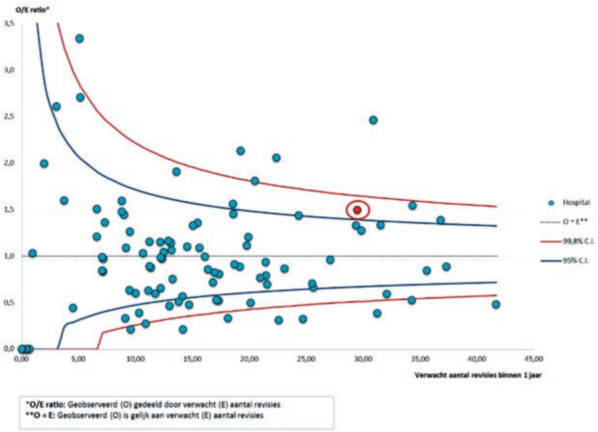

 Acta Orthopaedica 2020; 91 (DOI: 10.1080/17453674.2020.1827523)

a. Much worse than average (outside 99.8% control limit)
b. Worse than average (outside 95% control limit)
c. Average (within 95% control limit)
d. Better than average (outside 95% control limit)
e. Much better than average (outside 99.8% control limit)
f. I do not know

11. How does the department encircled in orange perform compared with the department encircled in red? (*This question involves part 2, see methods section survey in article*)

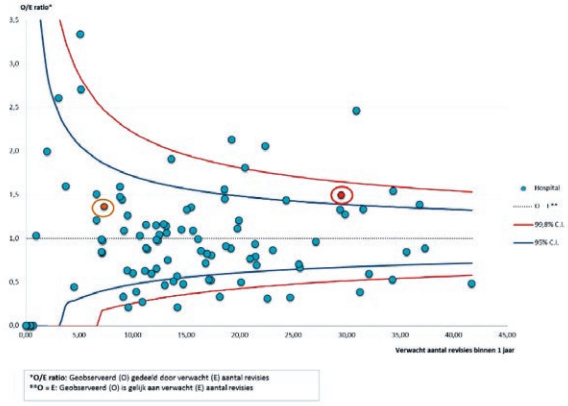

a. Better
b. Equal
c. Worse

Questions for all respondents: Awareness about performance of own healthcare center

12. Has your department, according to the funnel plots displayed on the LROI dashboard ever performed worse (above the 95% confidence interval) in the past two years? (*This question involves part 1, see methods section survey in article*)
   a. Yes ➞ Loop III (question 13, 14, 15)
   b. No ➞ Question 16
   c. I do not know ➞ Question 16

Loop III: Worse than average performance based on the LROI dashboard

13. Did you see in advance that your department performed worse than average? (*This question involves part 3, see methods section survey in article*)
   a. No,
   b. Yes, I felt this coming
   c. Yes, because we collect the same data as the LROI
   d. Yes, because my colleague drew my attention to this
14. Did you undertake quality improvement initiatives to improve? (*This question involves part 3, see methods section survey in article*)
   a. Yes, because we already knew what caused it
   b. Yes, after investigating the cause
   c. No, because the results may be due to coincidence and this is probably a one-off incident
   d. No, because in this period we were treating a relatively difficult patient population. The results will therefore improve automatically
   e. No, other reason, namely…
15. Did you use the LROI data to check whether the quality improvement initiative(s) have had effect(s)? (*This question involves part 3, see methods section survey in article*)
   a. No, we have not taken any action
   b. No, we did not check the effect of the intervention
   c. No, we introduced the intervention recently and are monitoring whether an effect is occuring
   d. Yes, the intervention(s) had no effect
   e. Yes, the intervention(s) had a positive effect

Questions for all respondents: Future improvements for feedback

16. Would you prefer to receive a signal earlier if the performance of your department improves or deteriorates compared with the national average? (*This question involves part 4, see methods section survey in article*)
   a. No, I can see that in the funnel plot on the LROI dashboard
   b. Yes, I would like an update every 6 months
   c. Yes, I would like an update every 3 months
   d. Yes, I would like an update every month
17. Which tabs on the LROI dashboard interest you the most? Put the results in order from most interesting (1) to least interesting (4) by dragging the "blocks". (*This question involves part 4, see methods section survey in article*)
   a. Total number of procedures performed
   b. 1-year revision rate
   c. PROMs
   d. Patient characteristics
18. Are there, in addition to the 1-year revision rates, in comparison with other healthcare centers, more outcomes in which you are interested (Yes/No)? You can check multiple options here. (*This question involves part 4, see methods section survey in article*)
   a. Prosthesis survival
   b. Improvement in PROMs postoperatively compared with preoperatively
   c. Length of hospital stay
   d. Hospital readmission
   e. Complications (other than revisions)
   f. None

19. How would you like to receive feedback on the outcomes of your department? (*This question involves part 4, see methods section survey in article*)
    a. Like the current situation, make selections on the LROI dashboard
    b. Make selections on a mobile application
    c. Tailored for my surgeon group, without making selections myself, on the LROI dashboard
    d. Tailored for my surgeon group, without making selections myself, on a mobile application
    e. Tailored for my surgeon group, without making selections myself, send by email
